# Uterine Microbiota of Dairy Cows With Clinical and Subclinical Endometritis

**DOI:** 10.3389/fmicb.2018.02691

**Published:** 2018-11-06

**Authors:** Meng-Ling Wang, Ming-Chao Liu, Jin Xu, Li-Gang An, Jiu-Feng Wang, Yao-Hong Zhu

**Affiliations:** Department of Veterinary Clinical Sciences, College of Veterinary Medicine, China Agricultural University, Beijing, China

**Keywords:** uterus, microbiota, uterine flush, cow, endometritis

## Abstract

The objective of this study was to characterize the uterine microbiota of dairy cows with clinical and subclinical endometritis and to identify the potential bacterial genera as well as their interactions associated with uterine disease. Uterine flush samples (*n* = 27) were collected from 13 healthy, 5 subclinical endometritic (SE), and 9 clinical endometritic (CE) cows at 30 days postpartum. Microbial DNA from uterine flush samples was subjected to sequencing of the 16S rRNA gene on the Illumina MiSeq platform. The uterine microbiota of healthy, SE, and CE cows had similarly complex microbial diversity, and shared 293 of 445 operational taxonomic units. However, endometritic and healthy cows could be discriminated by the relative abundance of bacterial genera. In CE cows, the uterine microbiota was characterized by increased abundance of *Fusobacterium* and unique presence of *Trueperella* and *Peptoniphilus*. For SE cows, known intrauterine pathogens were almost absent and the uterine microbiota was characterized by enrichment of *Lactobacillus* and *Acinetobacter*. Analysis of correlations between bacterial genera showed that the uterine microbiota exhibited two co-occurrence groups (i.e., the *Lactococcus* and the *Fusobacterium* COGs), indicating that the synergistic effect by co-occurred bacteria may be an important aspect of pathogenesis. Our findings support that common uterine pathogens are not associated with subclinical endometritis at 30 days postpartum and indicate the need of investigating the role of commensal bacteria such as *Lactobacillus*, and *Acinetobacter* in the inflammatory process of uterine endometrium.

## Introduction

Endometritis is one of the most important causes of infertility in dairy cows, resulting in high economic losses in the dairy industry (Sheldon et al., 2009; Wagener et al., 2017). Endometritis is a superficial inflammation of the endometrium without systemic signs (Sheldon et al., 2006). Clinical endometritis is defined as the presence of purulent or mucopurulent vaginal discharge at 21 or more days postpartum, accompanied by a prominent leukocyte infiltration into the uterine lumen. Subclinical endometritis is characterized by an increased proportion of polymorphonuclear neutrophils (PMN) cells in the endometrium, with the absence of signs of clinical endometritis (Kasimanickam et al., 2004). Indeed, a broad diversity of bacteria, including potential pathogens, can be observed in the uterus of 80–100% of dairy cows during the first 2 weeks postpartum (Sheldon et al., 2009). Depending on the balance between the immune response and uterine infection, about 25–40% of cows develop metritis within first 3 weeks postpartum (Markusfeld, 1987; Drillich et al., 2001); subsequently, 15–20% of cows develop clinical endometritis, and 30% develop subclinical endometritis beyond 3 weeks postpartum (LeBlanc et al., 2002; Gilbert et al., 2005; Cheong et al., 2011). Postpartum endometritis has a negative effect on reproductive performance as it delayed resumption of ovarian cycles, prolonged postpartum luteal phases, increased days to first service and days open, and decreased the conception rate (Kasimanickam et al., 2004; Ribeiro et al., 2013).

Studies using culture-dependent methods have identified several uterine pathogens associated with endometritis, including *Escherichia coli*, *Trueperella pyogenes*, *Fusobacterium necrophorum*, and *Prevotella* species (Dohmen et al., 1995; Williams et al., 2005; Carneiro et al., 2016). Members of the genera *Bacillus*, *Streptococcus*, and *Enterococcus*, in addition to coagulase-negative *Staphylococci*, are among the most frequently isolated intrauterine bacteria and have been described as potential or opportunistic pathogens (Wagener et al., 2014, 2015; Carneiro et al., 2016). Recently developed culture-independent molecular approaches based on sequencing have expanded our current knowledge of the uterine microbiome in cows with metritis, pyometra, and endometritis (Santos and Bicalho, 2012; Jeon et al., 2015; Knudsen et al., 2015, 2016; Bicalho et al., 2017a,b). *T. pyogenes* was the most important bacteriological risk factor for clinical endometritis, but not for subclinical endometritis (Prunner et al., 2014b). Bacterial growth density on the agar plates increased the risk for subclinical endometritis (Prunner et al., 2014a). Knowledge regarding the uterine microbiota in subclinical endometritic (SE) cows, mainly gleans from studies based on routine microbial isolation and culture techniques. The results from culture-based studies indicated that uterine infections with known pathogens play a minor role in SE cows (Sens and Heuwieser, 2013; Madoz et al., 2014; Prunner et al., 2014a,b).

In this study, we explored the uterine microbiota from the uterine flush samples of healthy, SE and clinical endometritic (CE) cows in an attempt to identify bacterial genera that were associated with endometritis via 16S rRNA gene profiling by high-throughput sequencing. Furthermore, we performed the co-occurrence network analysis to identity potential interactions between genera in the uterine microbiota of dairy cows. The data generated through this work might ultimately facilitate the development of efficient disease prevention and intervention strategies.

## Materials and Methods

### Experimental Design and Sampling

All animal procedures were performed in accordance with the approved guidelines and regulations, and the ethical approval of the Animal Ethics Committee of the China Agricultural University (CAU20140728-2).

The study was conducted on a commercial dairy farm in Beijing, China. The herd consisted of 800 milking Holstein dairy cows with an average milk production of 9,527 kg per lactation. A total of 38 cows were enrolled in the study. During the sample collection period, nine cows were excluded because of a systemic antibiotic treatment. Reasons for these antibiotic treatments were mastitis (*n* = 5), metritis (*n* = 2), pyometra (*n* = 1), vaginal lacerations (*n* = 1). Another two cows were excluded because of the poor quality of cytological smears. Therefore, the complete data set of 27 cows was used for statistical analyses. The average parity was 3.3, and an average body condition score (BCS) was 3.1 (see Supplementary Table S1). No differences were found among groups in parity and BCS. On day 30 postpartum, all cows underwent a vaginal inspection, rectal palpation of the uterus, endometrial cytological examination, and their overall condition was recorded. The BCS was evaluated on a scale from 1 to 5. The cows were selected for the study dependent on the health status of the uterus. Vaginal discharge was scored as previously described (Williams et al., 2005): score 0 with clear or translucent mucus; score 1 with mucus containing flecks of white or off-white pus; score 2 with less than 50% white or off-white mucopurulent material in the mucus; and score 3 with more than 50% purulent material, usually white or yellow, but occasionally sanguineous in the mucus. Cows exhibiting mucopurulent or worse (purulent or foul) vaginal discharge without signs of systemic illness as well as the presence of purulent material within the uterine lumen were classified as having clinical endometritis (*n* = 9). In the absence of purulent vaginal discharge, cows with the proportion of PMN ≥ 18% by cytological examination were classified as having subclinical endometritis (*n* = 5). Cows with a clear or translucent vaginal discharge that was not fetid or mucopurulent and with the proportion of PMN < 18% by cytological examination were classified as healthy (*n* = 13).

Uterine flush samples were collected from cows at 30 days postpartum. Uterus was flushed with saline, using a pipette (Santos and Bicalho, 2012). Briefly, each cow was restrained and the perineum area was disinfected with 70% ethanol. The infusion pipette covered with a protective plastic sheath was introduced into the cervix; the sheath was subsequently ruptured and the clean pipette tip was manipulated through the cervix into the uterus. The pipette has a deflated balloon in the tip. Once inside the uterus, the balloon was inflated to prevent vaginal or cervix contamination. A total of 30 mL of sterile saline was infused into the uterus, agitated gently, and a sample of the fluid aspirated. Recovered fluid was transferred to two polypropylene centrifuge tubes and placed on ice for transport to the laboratory within 4 h. One tube of each uterine flush sample was stored at -80°C for DNA extraction, the other tube was taken for cytological examination. Briefly, the uterine flush samples were centrifuged at 750 ×*g* for 10 min. After discarding the supernatant, the remaining pellets were re-suspended and smeared onto microscope slides. The slides were fixed and stained with Diff Quick. A total amount of 300 cells (endometrial epithelial cells and PMNs) were counted under a microscope by ×400 magnification to determine the proportion of PMN. A proportion of 18% PMN was set as the threshold for the diagnosis of subclinical endometritis in cows with clear vaginal discharge (Kasimanickam et al., 2004).

### DNA Extraction and Sequencing

Uterine flush samples collected from 27 cows were prepared for DNA extraction and sequencing of the 16S rRNA gene. An aliquot of 2 mL was centrifuged for 30 min at 15,000 ×*g* at 4°C. The supernatant was discarded, the pellets were suspended in 200 μL of phosphate-buffered saline (PBS) to concentrate microbial cells. The PBS suspension was used to isolate bacterial genomic DNA using a QIAamp DNA minikit (Qiagen, Valencia, CA, United States) according to the manufacturer’s protocol, with a minor modification: before AL buffer was added, samples were incubated with 400 mg of lysozyme for 12 h at 56°C to maximize bacterial DNA extraction. The purity and concentration of genomic DNA were determined using a spectrophotometer (Nanodrop 1000; Thermo Scientific, Waltham, MA, United States). Genomic DNA was amplified by PCR with primers that target the V3 and V4 hypervariable regions of the 16S rRNA gene. The forward primer sequence was 338F (5′-ACTCCTACGGGAGGCAGCAG-3′), and the reverse primer sequence was 806R (5′-GGACTACHVGGGTWTCTAAT-3′). An eight-base sequence unique to each sample preceded the primers for sample identification using a HotStarTaq Plus master mix kit (Qiagen) according to a custom Illumina preparation protocol. Amplicons were excised from 1.5% agarose gels and purified using the AxyPrep DNA Gel Extraction Kit (Axygen Biosciences, Union City, CA, United States) according to the manufacturer’s protocol and quantified using ST fluorometer (Promega, Madison, WI, United States). A composite sample library for sequencing was created by combining equimolar ratios of amplicons from the individual samples. The composite sample library was cleaned using an UltraClean-htp 96-well PCR cleanup kit (Mo Bio Laboratories, Carlsbad, CA, United States). Pooled amplicons were paired-end sequenced (PE 2 × 250) on an Illumina MiSeq platform according to standard protocols.

### Sequence Analysis

The sequence data were deposited in the NCBI Sequence Read Archive database (accession number SRP102408). Raw fastq files were demultiplexed and quality-filtered using the Quantitative Insights Into Microbial Ecology (QIIME) (Caporaso et al., 2010) with the following criteria: (i) The 250 bp reads were truncated at any site receiving an average quality score < 20 over a 50 bp sliding window, discarding the truncated reads that were shorter than 50 bp; (ii) there were exact barcode matching and a maximum of two nucleotide mismatches to primer sequences; (iii) no ambiguous bases; and (iv) only sequences that overlap longer than 10 bp were assembled according to their overlap sequence. Reads that could not be assembled were discarded. Chimeras were checked and excluded using the Uchime algorithm (version 4.2.40^1^ ). The resulting high-quality sequences were clustered into operational taxonomic units (OTUs) at 97% identity level using Usearch 6.1 methodology (version 6.1.544). All singleton OTUs were removed in an attempt to discard the majority of chimera sequences. The OTUs that reached at a 97% similarity level were used for alpha diversity, Good’s coverage, Venn diagram, rarefaction, and rank abundance curve analysis using Mothur (version 1.31.2). Sequences were subsampled to the lowest number of sequences found in all samples (9,666 reads) to evaluate alpha diversity. Alpha diversity was assessed by Shannon index, and the number of observed OTUs. The principal coordinate analysis (PCoA) based on Bray–Curtis distance was performed using OTUs from each sample and plotted by the vegan package in R.

Taxonomic classification of the representative sequence for each OTU was performed using the Ribosomal Database Project classifier^2^ (Release 11.1) with a cutoff of 80% homology against the Silva Gold reference database^3^(Release 128). OTUs were grouped at different levels of classification (phylum, class, order, family, and genus), at each level, unclassified OTUs were grouped together by the highest available resolution. A heat map was generated with average linkage hierarchical clustering of Bray–Curtis distance based on the relative abundances of genera per animal.

The correlations between the 28 most abundant genera were calculated using pairwise Spearman’s rank based on relative abundance in the R stats package. The correlation matrix was visualized and clustered in R using the Made4 package and Heatplot function, and hierarchical Ward-linkage clustering was used to define genus co-occurrence groups (COGs) (Biagi et al., 2016). The correlations were visualized in network interface with Cytoscape software (Shannon et al., 2003). The nodes represented genera, and the size of each node is proportional to the average relative abundance. The edges between nodes represented significant (*P* < 0.05) correlations, and the thickness of edge is proportional to the correlation strength.

### Statistical Analysis

Statistical analysis was carried out using R Statistical Language and GraphPad Prism (version 7.0) software. All continuous variables, such as relative abundance of bacteria, Shannon index, and the number of observed OTUs were analyzed using the Kruskal–Wallis analysis of variance on ranks, followed by Dunn’s test to adjust for multiple comparisons. Permutational multivariate analysis of variance (PERMANOVA) was performed using the vegan package in R. Correlations were calculated by Spearman’s rank correlation in the R stats package. The linear discriminant analysis (LDA) effect size (LEfSe)^4^ method was used to identify indicator bacteria differentiating the uterine microbiota between healthy and endometritic cows, which emphasizes both statistical significance and biological relevance. LEfSe uses the Kruskal–Wallis rank sum test with a normalized relative abundance matrix to detect differentially abundant features between groups and performs LDA to estimate the effect size of each feature (Segata et al., 2011). A significance level (alpha) of 0.05 and an effect size threshold of 3 were used for all indicators discussed. All tests for significance were two-sided, and a significance level of 0.05 was considered statistically significant in this study.

## Results

### Bacterial Diversity of the Uterine Microbiota

**FIGURE 1:**
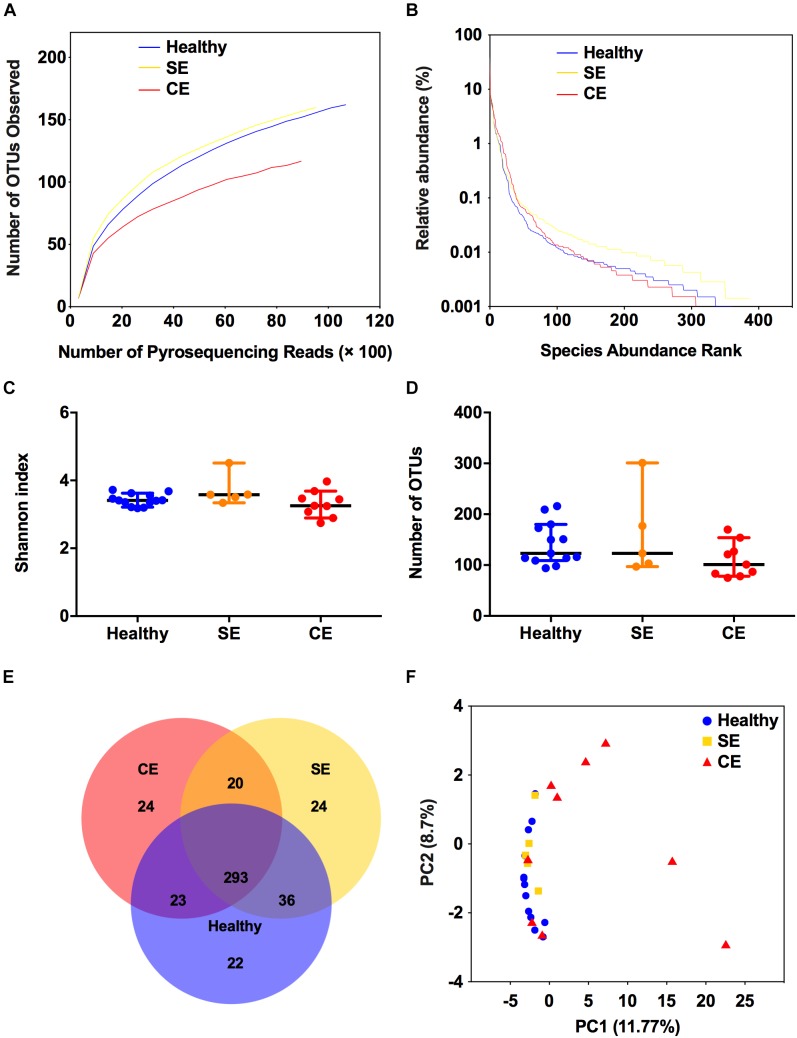
Structural comparison of the uterine microbiota. The rarefaction curves **(A)**, rank abundance curves **(B)**, Shannon index **(C)**, and the number of operational taxonomic units (OTUs) **(D)** were used to estimate alpha diversity of the uterine microbiota in healthy, SE, and CE cows. Symbols represent data from individual cows, data shown as median with 95% CI. **(E)** Venn diagram illustrating the common and exclusive OTUs in the uterine microbiota of the three groups. **(F)** Principal coordinate analysis based on the relative abundance of OTUs with Bray–Curtis distances, showing the differences between each individual cow. Healthy, healthy cows, *n* = 13; SE, subclinical endometritic cows, *n* = 5; CE, clinical endometritic cows, *n* = 9.

To profile the bovine uterine microbiota, we performed 16S rRNA sequencing for 27 uterine flush samples using an Illumina MiSeq platform. A total of 468,846 raw sequence reads were obtained. Following quality trimming and chimera checking, 392,246 high quality reads remained, accounting for 83.7% of the valid reads. The rarefaction curves (Supplementary Figure S1 and Figure 1A) showed that this sequencing depth was sufficient to cover the overall bacterial diversity. The value of Good’s coverage of each cow were greater than 99%, indicating that this sequencing method can characterize the true composition of uterine microbiota. The rank-abundance curves showed that a few species accounted for more than 1% abundance in the uterine microbiota (Figure 1B). Species diversity was measured as Shannon index, species richness was calculated as the number of OTUs. No differences were found among groups in species diversity and species richness (Figures 1C,D). Venn diagram showed that 293 of the 445 total OTUs were shared among groups (Figure 1E). These 293 shared OTUs dominated the uterine microbiota, represented 99.68%, 99.39%, and 98.67% of the total OTUs abundance in the healthy, SE, and CE cows, respectively. The uterine microbiota of healthy, SE, and CE cows had similar level of microbial diversity and shared most bacterial species (Figures 1C–E). The PCoA analysis showed that the samples from CE cows could be separated from healthy cows, although two CE samples were clustered with the healthy group (Figure 1F). The PERMANOVA analysis of the uterine samples showed significant differences in community composition between the healthy and CE groups (*P* = 0.004, *R*^2^ = 0.182), and no significant difference in community composition between the healthy and SE groups (*P* = 0.167, *R*^2^ = 0.097).

### Endometritis-Associated Alterations in the Uterine Microbiota

**FIGURE 2:**
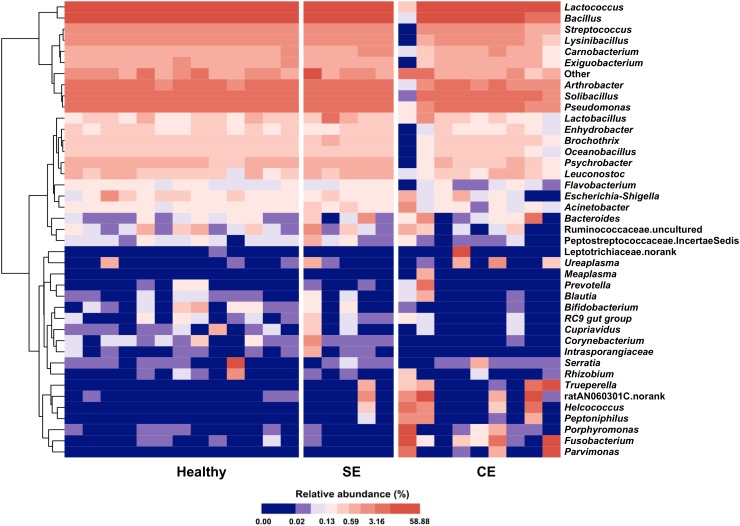
Heat map with average linkage clustering based on Bray–Curtis distance showing the relative abundances of the top 40 genera in each cow. The relative abundance of each genus is indicated by a gradient of color from blue (low abundance) to red (high abundance). Healthy, healthy cows, *n* = 13; SE, subclinical endometritic cows, *n* = 5; CE, clinical endometritic cows, *n* = 9.

A total of 17 phyla were identified in the uterine microbiota of all samples. Taxonomic assignment showed that the Firmicutes (76.7%), Proteobacteria (8.1%), Actinobacteria (5.9%), Bacteroidetes (4.6%), Fusobacteria (4.3%), and Tenericutes (0.2%) were the six most abundant phyla in the uterus of all dairy cows, accounting for 99.8% of the total abundance. In total, 206 genera were identified across all the samples. A heat map of the 40 most abundant genera (at 0.1% or greater abundance within either group) is shown in Figure 2, accounting for 99.66%, 99.48%, and 98.61% of the total genera abundance in the healthy, SE, and CE groups, respectively. *Lactococcus*, *Bacillus*, *Solibacillus*, *Pseudomonas*, and *Arthrobacter* were the five most abundant genera with little variations in the uterus of healthy and SE cows (Supplementary Table S4). In CE cows, the *Lactococcus*, *Bacillus*, *Fusobacterium*, ratAN060301.norank, and *Solibacillus* were the top five abundant genera. The genus-level heat map analysis showed an obvious shift in the uterine microbiota of CE cows, with an increase in the genera *Fusobacterium*, *Parvimonas*, *Porphyromonas*, *Peptoniphilus*, *Helcococcus*, *Trueperella*, and ratAN060301.norank (OTU154, uncultured *Porphyromonas* species in the family ratAN060301, order Bacteroidales), compared with healthy and SE cows.

**FIGURE 3:**
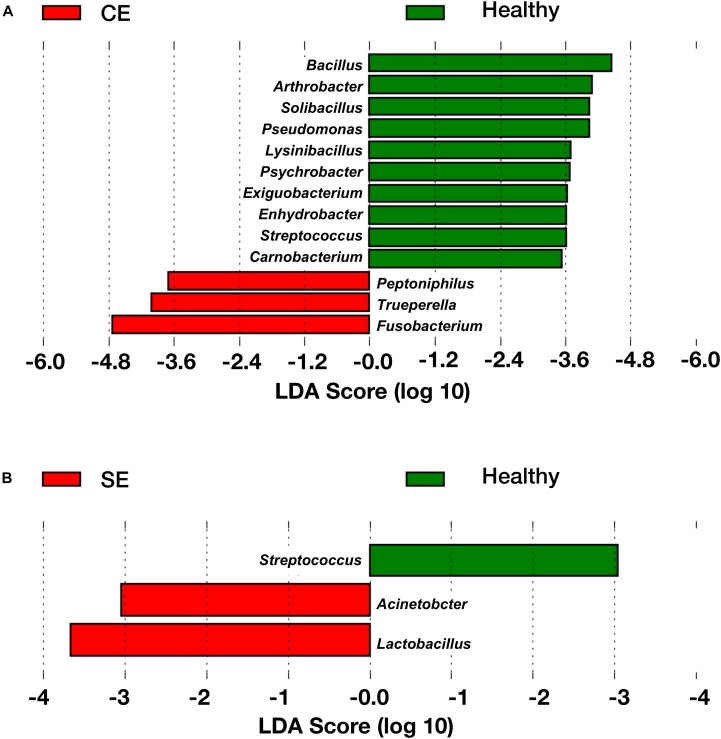
The linear discriminant analysis (LDA) effect size plots showing the differences in the uterine microbiota between healthy cows and CE cows **(A)**, and between healthy cows and SE cows **(B)**. The histogram shows the LDA effect size computed for features at genus level. Healthy-enriched genera are indicated with positive LDA scores (green), and genera enriched in CE or SE cows are indicated with negative LDA scores (red). Only genera meeting a significant level of 0.05 and an effect size threshold of 3 are plotted. Healthy, healthy cows, *n* = 13; SE, subclinical endometritic cows, *n* = 5; CE, clinical endometritic cows, *n* = 9.

To identify genera associated with endometritis, LEfSe was performed using the 28 most abundant bacterial genera in the uterine microbiota of healthy and endometritic cows. Compared with healthy cows, *Fusobacterium*, *Trueperella*, and *Peptoniphilus* were discriminately enriched (LDA sores > 3.5) in CE cows (Figure 3A and Supplementary Table S2). In SE cows, *Lactobacillus*, and *Acinetobacter* were discriminately increased (LDA scores > 3), compared with healthy cows (Figure 3B and Supplementary Table S3).

**FIGURE 4:**
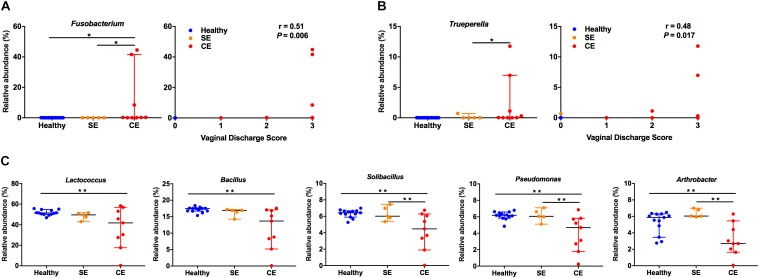
The relative abundance distribution of *Fusobacterium* **(A)**, *Trueperella* **(B)** among groups, and correlations between vaginal discharge scores. Cows were assigned to four groups according to vaginal discharge score: 0, clear or translucent mucus; 1, clear discharge with flecks of pus; 2, mucopurulent, not fetid discharge; 3, purulent or fetid discharge. *r*, Spearman’s coefficient. **(C)** Analysis of the relative abundance of *Lactococcus*, *Bacillus*, *Solibacillus*, *Pseudomonas*, and *Arthrobacter* in healthy, SE and CE cows by Kruskal–Wallis rank-sum test. Symbols represent data from individual cows, data shown as median with 95% CI. Healthy, healthy cows, *n* = 13; SE, subclinical endometritic cows, *n* = 5; CE, clinical endometritic cows, *n* = 9. ^∗^*P* < 0.05, ^∗∗^*P* < 0.01.

To further explore the relationship between specific bacteria and clinical endometritis, we stratified cows by vaginal discharge score as a clinical sign and performed a Spearman’s rank correlation tests on the relative abundances of the 28 most abundant bacterial genera. The relative abundance of *Fusobacterium* increased with increasing vaginal discharge score (Spearman’s *r_s_* = 0.51, *P* = 0.006) (Figure 4A). Likewise, the relative abundance of *Trueperella* increased with increasing vaginal discharge score (Spearman’s *r_s_* = 0.48; *P* = 0.017) (Figure 4B).

We also compared the relative abundances of the 28 most abundant genera in the uterine microbiota using Kruskal–Wallis rank-sum test (Figure 4 and Supplementary Table S3). Compared with healthy cows, the relative abundance of *Fusobacterium* increased in CE cows, the abundance of *Lactococcus*, *Bacillus*, *Solibacillus*, *Pseudomonas*, and *Arthrobacter* decreased (*P* < 0.05). *Lactobacillus* and *Acinetobacter* were more abundant in SE cows than in healthy cows (*P* < 0.05). The abundance of *Fusobacterium* and *Trueperella* decreased in SE cows, compared with those in CE cows (*P* < 0.05). No differences in the abundance of *Lactococcus*, *Bacillus*, *Solibacillus*, *Pseudomonas*, and *Arthrobacter* were observed between the healthy and SE cows.

### Interactions Between Bacterial Genera in the Uterine Microbiota

**FIGURE 5:**
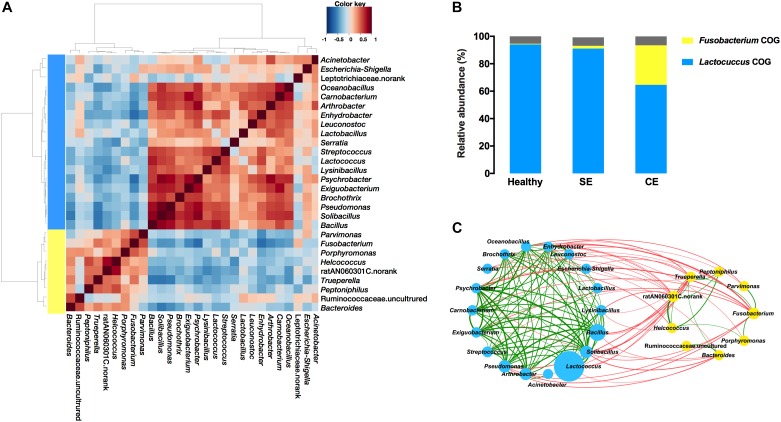
Co-occurrence groups (COGs) assignment depended on heat plot **(A)** showing Spearman correlations between genera, clustered by Ward-linkage hierarchical clustering. **(B)** The average cumulative abundance of genera in each COG for healthy, SE and CE cows is also plotted. Colors are indicative of the two identified COGs: *Lactococcus* COG (blue) and *Fusobacterium* COG (yellow). The proportion of genera not considered in the COG assignment is colored gray. **(C)** The network plot showing correlations between bacterial genera in the uterine microbiota of all cows **(C)**. The nodes represent the genera; the circle size of each node is proportional to its average relative abundance in all samples. Blue and yellow nodes indicate genera belong to *Lactococcus* COG and *Fusobacterium* COG, respectively. The edges between nodes represent significant (*P* < 0.05) correlations between genera, the thickness of edge is proportional to the correlation strength (see Supplementary Table S5). Green and red edges indicate positive and negative correlations, respectively. Healthy, healthy cows, *n* = 13; SE, subclinical endometritic cows, *n* = 5; CE, clinical endometritic cows, *n* = 9.

We evaluated the correlations between the 28 most abundant bacterial genera, and performed hierarchical Ward-linkage clustering of the correlations to define co-occurrence group (COG) (Figure 5A). The distribution of COGs differed significantly among groups, determined by PERMANOVA analysis using the Bray–Curtis dissimilarity (*Fusobacterium* COG *F* = 2.31, *P* = 0.003; *Lactococcus* COG *F* = 2.39, *P* = 0.002). The *Lactococcus* COG represented the majority of the uterine microbiota in terms of high predominate abundance, accounted for 94.1%, 91.1%, and 66.1% in the healthy, SE, and CE groups, respectively. The *Fusobacterium* COG (accounted for 28.39%) was exclusively enriched in the uterine microbiota of CE cows (Figure 5B). *Fusobacterium*, *Porphyromonas*, *Trueperella*, *Helcococcus*, and *Peptoniphilus* represented 10.58%, 3.05%, 2.24%, 1.55%, and 0.66% of the total bacterial population in CE cows, while little abundance was found in the uterine microbiota of healthy and SE cows (representing less than 0.2% of the total bacterial population) (Supplementary Table S4). At species level, sequences from *F. necrophorum* (OTU430), *T. pyogenes* (OTU99), *Helcococcus ovis* (OTU311) and *Peptoniphilus indolicus* (OTU295) were identified in this study (Supplementary Table S5).

A number of positive correlations were found among genera from the same COG (Figure 5C and Supplementary Table S6). Major uterine pathogens such as *Fusobacterium*, *Trueperella*, *Bacteroides*, and *Porphyromonas* along with other uterine pathogens such as *Peptoniphilus* and *Helcococcus* belonged to the *Fusobacterium* COG, and had positive correlations. *Fusobacterium* showed a positive correlation with *Trueperella* (Spearman’s *r_s_* = 0.43, *P* = 0.026), which was positively correlated (*P* < 0.05) with *Peptoniphilus*, *Helcococcus*, ratN060301C.norank, and *Bacteroides*. *Helcococcus* and ratN060301C.norank were positively correlated (*P* < 0.05) with *Peptoniphilus. Helcococcus* was also positively correlated (*P* < 0.05) with ratN060301C.norank and *Porphyromonas. Bacteroides* was also positively correlated (*P* = 0.002) with Ruminococcaceae uncultured. *Fusobacterium* showed a positive correlation with *Parvimonas* (*P* = 0.003). Within the *Lactococcus* COG, *Arthrobacter* was positively correlated with *Lactobacillus* (Spearman’s *r_s_* = 0.56, *P* = 0.018), and with *Psychrobacter* (Spearman’s *r_s_* = 0.80, *P* = 0.00004). *Arthrobacter*, *Psychrobacter*, *Bacillus*, *Solibacillus*, and *Pseudomonas* were positively correlated with many bacteria including *Psychrobacter*, *Carnobacterium*, *Exiguobacterium*, and *Brochothrix.*

Negative correlations were found between genera from the *Lactococcus* COG and the *Fusobacterium* COG (Figure 5C and Supplementary Table S6). The abundance of *Fusobacterium* was negatively correlated with the abundance of *Arthrobacter* (Spearman’s *r_s_* = -0.53, *P* = 0.028), and with the abundance with *Psychrobacter* (Spearman’s *r_s_* = -0.64, *P* = 0.0003). *Psychrobacter, Bacillus*, *Solibacillus*, and *Pseudomonas* were negatively (*P* < 0.05) correlated with *Trueperella*. Genera with negative correlations with *Fusobacterium* and*/*or *Trueperella*, comprising *Arthrobacter*, *Psychrobacter*, *Bacillus*, *Solibacillus*, and *Pseudomonas*, were positively correlated (*P* < 0.05) with many other bacteria including *Psychrobacter*, *Carnobacterium*, *Exiguobacterium*, and *Brochothrix.*

## Discussion

In the present study, we investigated the disease-related alterations as well as bacteria interactions in the uterine microbiota of CE and SE cows. Previous studies have described the diversity and complexity of the bacterial community in the postpartum uterus of dairy cows (Santos and Bicalho, 2012; Jeon et al., 2015; Knudsen et al., 2015, 2016; Bicalho et al., 2017a,b). Here, we performed co-occurrence analysis to obtain a comprehensive understanding of the complex bacterial interactions in bovine uterus.

We demonstrated that *Fusobacterium*, *Trueperella*, and *Peptoniphilus* were associated with clinical endometritis, and *Fusobacterium* and *Trueperella* were positively correlating with purulent vaginal discharge. Notably, CE-associated genera *Fusobacterium*, *Trueperella*, and *Peptoniphilus*, along with other pathogens such as *Porphyromonas*, *Parvimonas*, *Bacteroides*, and *Helcococcus* were found to belong to the *Fusobacterium* COG, and had positive correlations. Therefore, it is likely that *Fusobacterium* acts synergistically with *Trueperella*, *Porphyromonas*, *Parvimonas* and other bacteria, to cause dysbiosis in the uterine microbiota of CE cows. *F. necrophorum*, and *T. pyogenes* have been recognized as the major uterine pathogens associated with metritis, endometritis and purulent vaginal discharge (Dohmen et al., 1995; Williams et al., 2005; Bicalho et al., 2012; Prunner et al., 2014b). It has been proposed that *Trueperella* and the Gram-negative anaerobes *Fusobacterium*, *Bacteroides*, and *Porphyromonas* act synergistically to cause metritis and endometritis in the uterus (Bonnett et al., 1991; Bicalho et al., 2012; Prunner et al., 2014b). *T. pyogenes* causes cytolysis in the endometrium by secreting pyolysin (Amos et al., 2014). *F. necrophorum* produces leukotoxins (Nagaraja et al., 2005), *Bacteroides* produces short-chain fatty acids (Rotstein, 1993), and *Porphyromonas levii* produces an immunoglobulin protease that inhibits phagocytosis (Lobb et al., 1999). It is widely believed that *T. pyogenes* support *F. necrophorum* growth and colonization by producing an unknown growth factor (Dadarwal et al., 2017). A synergy between *F. necrophorum* and *Porphyromonas levii* has been hypothetically suggested for their co-localization in the lamina propria of the uterus (Karstrup et al., 2017a). It is therefore plausible that uterine pathogens might assist each other in avoiding uterine defense mechanisms and interact to facilitate colonization of the endometrium. Similar cooperative interactions between pathogens were also observed in cows with metritis or purulent vaginal discharged (Bicalho et al., 2012, 2017a,b; Jeon et al., 2015, 2017). Pathogenic bacteria (such as *Trueperella* spp., *Fusobacterium* spp.) were also present in the uterus of virgin heifers and of pregnant cows (Karstrup et al., 2017b,c; Moore et al., 2017). Collectively, the co-occurrence of uterine pathogens could be considered of major importance in the development of uterine infection. The cooperative interspecies signaling and mechanism behind synergisms need to be elucidated.

Contrast with clinical endometritis, cows with subclinical endometritis harbored a small proportion of the *Fusobacterium* COG, constituting only 1.5% of the total number of sequences. *Fusobacterium*, *Trueperella* were rarely detected in samples from SE cows. Early culture-based study demonstrated that subclinical endometritis at 21 days postpartum was not associated with *T. pyogenes* (Prunner et al., 2014a). Early culture-based study also found that *T. pyogenes* were frequently isolated from CE cows, and no bacteria were isolated from SE cows (Madoz et al., 2014). Our observations by sequencing of 16S rRNA gene corroborate previous observations, supporting that uterine infections with major pathogens play a minor role in SE cows compared with CE cows. The establishment of uterine infections depends on the pathogenicity of invading bacteria and the local immune state. Many factors influence bacteria pathogenicity, including bacterial load, various strains, bacterial virulence factors, and interactions between species etc. (Dadarwal et al., 2017). PMN infiltration into the uterine lumen as an indicator of subclinical endometritis is associated with increased endometrial mRNA expression of pro-inflammatory mediators, including cytokines antimicrobial peptides, acute phase proteins and prostaglandins (Fischer et al., 2010; Brodzki et al., 2015). Brodzki et al. (2015) hypothesized that high levels of IL-10 in SE cows contribute to a weakened local immune response in the endometrium, leading to persistent uterine inflammation in the postpartum period (Brodzki et al., 2015). Metabolic imbalances also increase the risk of subclinical endometritis, particularly a negative energy balance, which interferes with an adequate immune response (Wagener et al., 2017). Elevated concentrations of non-esterified fatty acids and beta-hydroxybutyric acid, and a poor BCS increase the risk for subclinical endometritis (Galvão et al., 2010; Heidarpour et al., 2012).

The uterine microbiota of SE cows was characterized by enrichment of *Lactobacillus* and *Acinetobacter*. Intravaginal administration of certain strains of *Lactobacillus* reduced the incidence of uterine diseases in treated cows with enhanced secretory immunoglobulin A production in the vaginal mucus (Deng et al., 2015). *Lactobacillus* species isolated from the bovine uterus, such as *L. amylovorus* and *L. ruminis*, stimulate an immune response without cytotoxic effects (Gärtner et al., 2015). *Lactobacillus rhamnosus* GR-1 reduces *E. coli*-induced release of pro-inflammatory cytokines in primary bovine endometrial epithelial cells *in vitro* (Liu et al., 2016). *Acinetobacter* spp. are observed in the environment (e.g., soil and water) and present in the microbiota of healthy human skin, cattle udder skin, and cattle gut (Yeoman et al., 2018). *A. baumannii* is the most important species, since it causes serious infections in human (De Amorim and Nascimento, 2017). *Acinetobacter* strains isolated from samples of milk and milk derivatives could be opportunistic pathogens (Gurung et al., 2013). *Acinetobacter* strains can be isolated from intrauterine samples collected from repeat breeder cows (Pothmann et al., 2015). Due to the limited information of 16S rRNA sequencing method, it is difficult to define pathogenic strains or harmless commensals associated with subclinical endometritis. In this study, whether these bacterial changes are a cause or a consequence of uterine inflammation is uncertain. Our findings increase the current knowledge of the uterine microbiota in SE cows and provide a basis for future detailed *in vitro* studies to decipher the affect of these bacteria on immune response in uterine endometrium.

The *Fusobacterium* COG had negatively correlations with the *Lactococcus* COG and dramatically more abundant in CE cows, implying that there might be local colonization resistance between the *Fusobacterium* COG and *Lactococcus* COG, linked to the microbiota dysbiosis in CE cows. Accordingly, the overgrowth of the *Lactococcus* COG may result in a decrease of the *Fusobacterium* COG. Therefore, competition between members of the *Fusobacterium* COG and *Lactococcus* COG might restrict the overgrowth of potential pathogens. The composition and function of microbial communities is thought to be largely shaped by interspecies competition for the available resources (Zengler and Zaramela, 2018). The cooperative interactions among co-occurring bacteria, such as metabolite exchanges, could promote group survival under nutritionally challenging conditions (Zelezniak et al., 2015). The different uterine bacterial composition between healthy and diseased cows likely reflect the differing nutritional and physiological conditions. Identifying the source of critical nutrients that support pathogenic overgrowth will be crucial to increasing our understanding of disease pathogenesis and could potentially assist in developing novel treatment strategies (Zengler and Zaramela, 2018). Such competitive and cooperative interactions and mechanisms of establishing infection warrants further detailed studies.

Investigation the uterine microbiota in uterine flush samples from postpartum dairy cows by high throughput sequencing of 16S rRNA gene showed that major uterine pathogens such as *Fusobacterium*, *Trueperella*, and *Peptoniphilus* were enriched in the uterine microbiota of CE cows, but were almost absent in the uterine microbiota of SE cows. Our results demonstrated that known uterine pathogens had co-existence relationships with other bacteria, such as *Porphyromonas*, *Bacteroides*, *Helcococcus*, and *Parvimonas*, suggesting that their synergistic effects may be crucial contributors in uterine infection. Our findings support that major uterine pathogens are not associated with subclinical endometritis at 30 days postpartum and indicate the need of investigating the role of commensal bacteria such as *Lactobacillus*, and *Acinetobacter* in the inflammatory process of uterine endometrium.

## Author Contributions

M-LW, M-CL, Y-HZ, and J-FW conceived and designed the experiments. M-LW, M-CL, JX, and L-GA performed the experiments. M-LW performed sequencing analysis and wrote the manuscript.

## Conflict of Interest Statement

The authors declare that the research was conducted in the absence of any commercial or financial relationships that could be construed as a potential conflict of interest.

## Footnotes

1 http://drive5.com/usearch/manual/uchime_algo.html

2 http://rdp.cme.msu.edu/

3 http://www.arb-silva.de

4 http://huttenhower.sph.harvard.edu/lefse/

## Supplementary Material

FIGURE S1 Rarefaction curves of each individual cow. Healthy, healthy cows, *n* = 13; SE, subclinical endometritic cows, *n* = 5; and CE, clinical endometritic cows, *n* = 9.

TABLE S1 The basic information of cows included in this study.

TABLE S2 Key features are statistically different between the uterine microbiota of healthy and clinical endometritic cows.

TABLE S3 Key features are statistically different between the uterine microbiota of healthy and subclinical endometritic cows.

TABLE S4 Relative abundance of the 28 most abundant genera detected in the uterine of postpartum healthy and endometritic dairy cows at 30 days postpartum.

TABLE S5 Relative abundance of 28 most abundant species detected in the uterine of postpartum healthy and endometritic dairy cows at 30 days postpartum.

TABLE S6 Correlations among the 28 most abundant genera detected in the uterus of 27 cows at 30 days post parturition. Only significant (*P* < 0.05) Spearman coefficients of correlation are presented.
