# Supplementary figures and images for: Uterine Microbiota of Dairy Cows With Clinical and Subclinical Endometritis
Source: Front Microbiol. 2018 Nov 6;9:2691. doi: 10.3389/fmicb.2018.02691 (PMC6232309; doi:10.3389/fmicb.2018.02691)

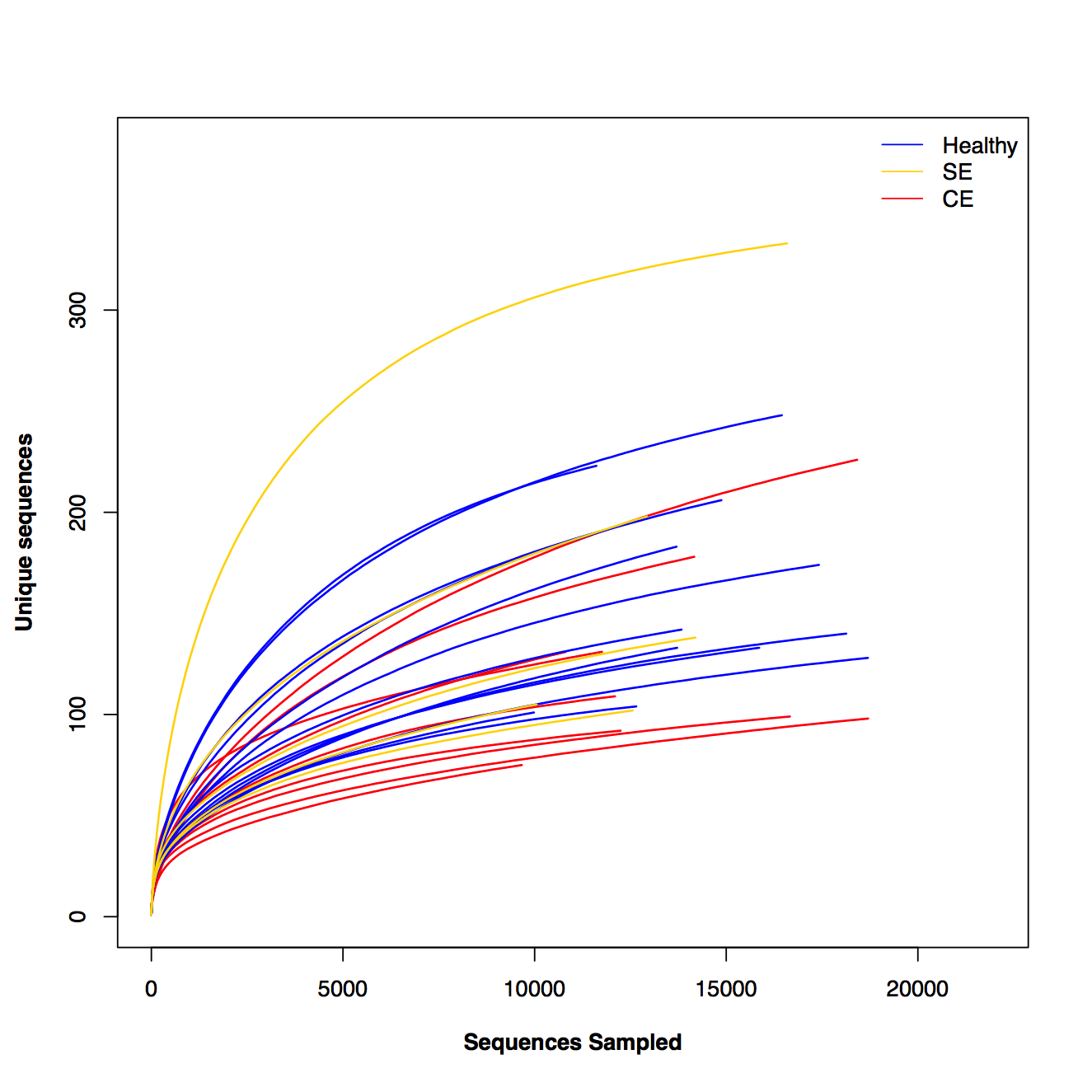

Supplement: FIGURE S1 — Rarefaction curves of each individual cow. Healthy, healthy cows, n = 13; SE, subclinical endometritic cows, n = 5; and CE, clinical endometritic cows, n = 9. [file Image_1.tiff]
